# Unprotected sidewalls of implantable silicon-based neural probes and conformal coating as a solution

**DOI:** 10.1038/s41529-021-00154-9

**Published:** 2021-02-10

**Authors:** Pejman Ghelich, Nicholas F. Nolta, Martin Han

**Affiliations:** 1 Department of Biomedical Engineering, University of Connecticut, Storrs, CT, USA; 2 Institute of Materials Science, University of Connecticut, Storrs, CT, USA

## Abstract

Silicon-based implantable neural devices have great translational potential as a means to deliver various treatments for neurological disorders. However, they are currently held back by uncertain longevity following chronic exposure to body fluids. Conventional deposition techniques cover only the horizontal surfaces which contain active electronics, electrode sites, and conducting traces. As a result, a vast majority of today’s silicon devices leave their vertical sidewalls exposed without protection. In this work, we investigated two batch-process silicon dioxide deposition methods separately and in combination: atomic layer deposition and inductively-coupled plasma chemical vapor deposition. We then utilized a rapid soak test involving potassium hydroxide to evaluate the coverage quality of each protection strategy. Focused ion beam cross sectioning, scanning electron microscopy, and 3D extrapolation enabled us to characterize and quantify the effectiveness of the deposition methods. Results showed that bare silicon sidewalls suffered the most dissolution whereas ALD silicon dioxide provided the best protection, demonstrating its effectiveness as a promising batch process technique to mitigate silicon sidewall corrosion in chronic applications.

## INTRODUCTION

A wide variety of implantable neural devices have been developed using microfabrication as a reliable and reproducible method for producing large batches of electrical^1,2^, chemical^3^, optical^4^, multimodal^5^, and microfluidic devices^6^. These devices have expanded our knowledge about the nervous systems and may soon have clinical applications in treating neurological disorders^7^. In particular, an early example of a device with significant impact was the Michigan probe^1^. Other groups have since innovated on this design style^8,9^, adding features such silicon-on-insulator (SOI) shank shaping^10–13^, high channel counts^14–16^, and CMOS-based microelectrodes^17–19^. The logical next step is to translate these technologies to clinical applications. While there have been successful long-term chronic studies in animals^12,13,20^, to date, there has been no U.S. Food and Drug Administration (FDA) clinical approval for “multisite” (multiple microelectrodes per shank) silicon-based microelectrodes. This stands in contrast to the Utah Electrode Array or Blackrock Array, which has been approved for human implantation for up to one month but is limited to only one electrode site at the tip of each shank^21^. For penetrating multisite microelectrodes and other silicon-based devices of various modalities to be approved for human use, long-term reliability and biostability must be demonstrated.

Microelectronics implanted in vivo are typically protected by layers of chemically inert, electrically insulating materials such as silicon dioxide (SiO_2_)^4,22^, silicon nitride (SiN_x_)^11^, multilayer stacks of SiO_2_ and SiN_x_^13^, or polymers such as Parylene C^13,16^, polyimide^23^, or SU-8^24^. These materials prevent exposure of conducting elements to the body environment, which can compromise device performance by creating short circuits and allowing corrosion to occur^22,25^. Although the top surfaces are protected, almost all penetrating multisite silicon-based devices leave the sidewalls of the silicon shanks exposed to body fluids without protection. This is generally considered acceptable as the sidewalls do not have conducting elements on them. However, silicon corrodes in saline environments at a rate depending on the temperature, chemistry of the solution, and silicon doping level. In a study by Hwang et al., their most physiologically relevant measurement was 36.8 μm per year for undoped silicon in 37 °C bovine serum^26^. In another study, they showed that heavily doping the silicon slowed dissolution by about an order of magnitude^27^. Unfortunately, for microscale devices intended to be implanted for decades, even a few microns of dissolution per year can contribute to device failure^22,25^. Therefore, unprotected silicon sidewalls may present a barrier to clinical approval for permanent implantation and neural stimulation applications.

With the exceptions of single-tip devices such as microwires and Blackrock array, only one study, to our knowledge, attempted to protect the sidewalls. In this previous study, Parylene C was deposited as a conformal coating after releasing the silicon probes^13^. However, this method required serial laser ablation to expose the electrode sites, with a narrow parameter space between successful removal of Parylene and damage to the underlying electrode material. An ideal sidewall protection technique would be batch process-compatible and utilize a low-temperature process that does not preclude the use of commonly used metals (e.g., gold, titanium) and photoresist.

In this work, we explored two deposition techniques that met these constraints: atomic layer deposition (ALD) and inductively-coupled plasma chemical vapor deposition (ICPCVD). Both these processes are compatible with batch processing and are performed at photoresist friendly temperatures (≤130 °C), which allows us to use photoresist to protect top device features. For the material, we chose SiO_2_ due to its biocompatibility and biostability^28–30^. In order to assess the sidewall coverage, we utilized wet etching using potassium hydroxide (KOH) as a silicon etchant. KOH has high etch selectivity for silicon over SiO_2_^31,32^, so any pinholes or defects in the sidewall protection will allow rapid etching of the underlying silicon, making this a straightforward, rapid, and simple method to evaluate the coverage quality. Although concentrated KOH is not present in the physiological environment, identifying pinholes and defects is useful, since these spots are likely to be weak points for degradation of silicon in vivo. We fabricated test devices with the same shape and material composition as our previously-validated neural microelectrodes^12,13,20^, and coated the sidewalls of the shanks with ALD SiO_2_, ICPCVD SiO_2_, both, or neither (control). To compare the success of each technique, we used focused ion beam (FIB) cross sectioning, scanning electron microscopy (SEM), and 3D extrapolations of etched silicon volumes.

## RESULTS

### Qualitative observation of exposed silicon surface over time

**Fig. 1:**
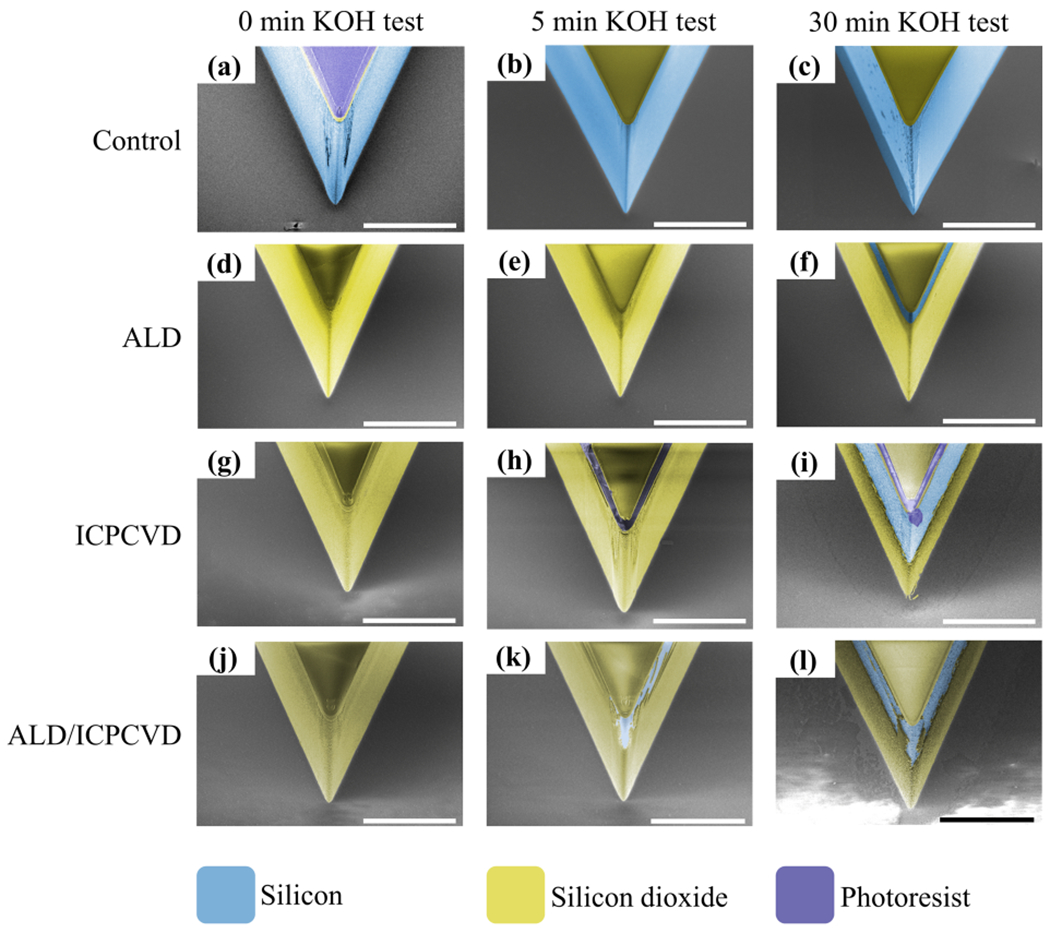
Front view SEMs of device tips showing progressive etching of the silicon and SiO_2_ sidewalls during the KOH etch test. The columns correspond to etch durations of 0 min, 5 min, and 30 min, while the rows represent deposition methods. False coloring indicates different materials. The less exposed silicon (blue) on the sidewalls, the better the protection. The scale bars are 50 μm. The control group (top row, **a–c**) suffered the most etching whereas the ALD film (second row, **d–f**) provided the best protection. The ICPCVD (third row, **g–i**) and ALD/ICPCVD (fourth row, **j–l**) films showed obvious etching over time. Partial exposure of silicon through SiO_2_ is visible in (**f**), (**i**), (**k**), and (**l**).

As shown in Fig. 1, the front views of the device tips revealed the etched profiles of the sidewalls before and after KOH wet etching. The first row (Fig. 1a–c) shows a control device (bare silicon). After 30 min in KOH, silicon was extensively etched, as expected. The second row (Fig. 1d–f) shows that ALD SiO_2_ was intact after 5 min, and even after 30 min, with only a thin band of exposed silicon visible near the top edge. The third row (Fig. 1g–i) shows ICPCVD sidewall protection which, after 5 min of etching, kept the silicon mostly intact (Fig. 1h). However, after 30 min, roughly half of the silicon wall was exposed (Fig. 1i). The fourth row (Fig. 1j–l) shows the dual ALD and ICPCVD SiO_2_ films. After 5 min of etching, the sidewall protection films were partially collapsed near the top surface (Fig. 1k), and after 30 min, more silicon was exposed (Fig. 1l), but still less severe than ICPCVD alone (Fig. 1i). The spatial pattern of the insulation breakdown was more irregular than the other groups.

### Quantification of etched silicon volume and overall assessment

**Fig. 2:**
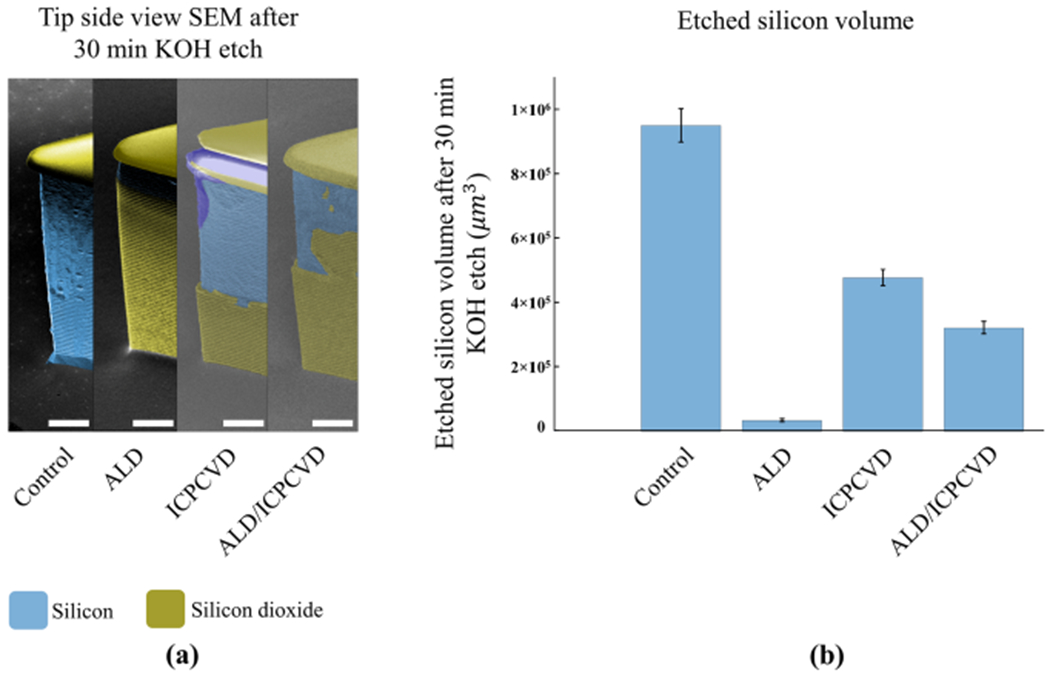
Quantification of etched silicon volume after 30 min KOH etching. **a** Side view SEM images, as noted in Fig. 6a, from each group after 30 min KOH etching. False coloring is identical to Fig. 1. Bright purple areas on ICPCVD panel are charged up photoresist during imaging. The scale bars are 10 μm. **b** Comparison of etched silicon volume for each group using 3D extrapolated models. ALD-protected devices showed the least loss of silicon sidewalls whereas bare silicon was etched the most. The error bars are standard deviation of the mean for each group (*>N* = 3). Tukey pairwise comparisons confirmed significant differences for all combinations after one-way ANOVA (*p* < 0.01 and 95% confidence level).

Quantification of the etched silicon volume was performed based on the side view SEM images, as shown in Fig. 2a. Extrapolated 3D volumes were quantified and plotted in Fig. 2b, enabling comparison among the four sample groups. Control devices were heavily etched along the entire sidewall surface by an average volume of 952,381 ± 52,662 μm^3^. ALD-protected devices, meanwhile, had the least amount of silicon etched: 33,682 ± 2925 μm^3^. Average etched volume for ICPCVD and ALD/ICPCVD were 477,898 ± 23,383 μm^3^ and 322,052 ± 18,510 μm^3^, respectively. One-way ANOVA showed there was a significant difference among the means of etched volumes (*p* < 0.01) and Tukey pairwise comparisons confirmed significant differences for all combinations with 95% confidence. Overall, ALD resulted in the least etched volume and was, therefore, the best protection, followed by ALD/ICPCVD, then ICPCVD. Surprisingly, this means that ALD outperformed ALD/ICPCVD, suggesting that ICPCVD after ALD actually reduced the performance of the ALD layer. In contrast, ALD did not reduce the performance of ICPCVD, because ALD/ICPCVD outperformed ICPCVD. In this study, the residual stress for ICPCVD film was measured at 32.88 ± 1.58 MPa, compressive.

### High-resolution SEM analysis of ALD/ICPCVD failure mode

**Fig. 3:**
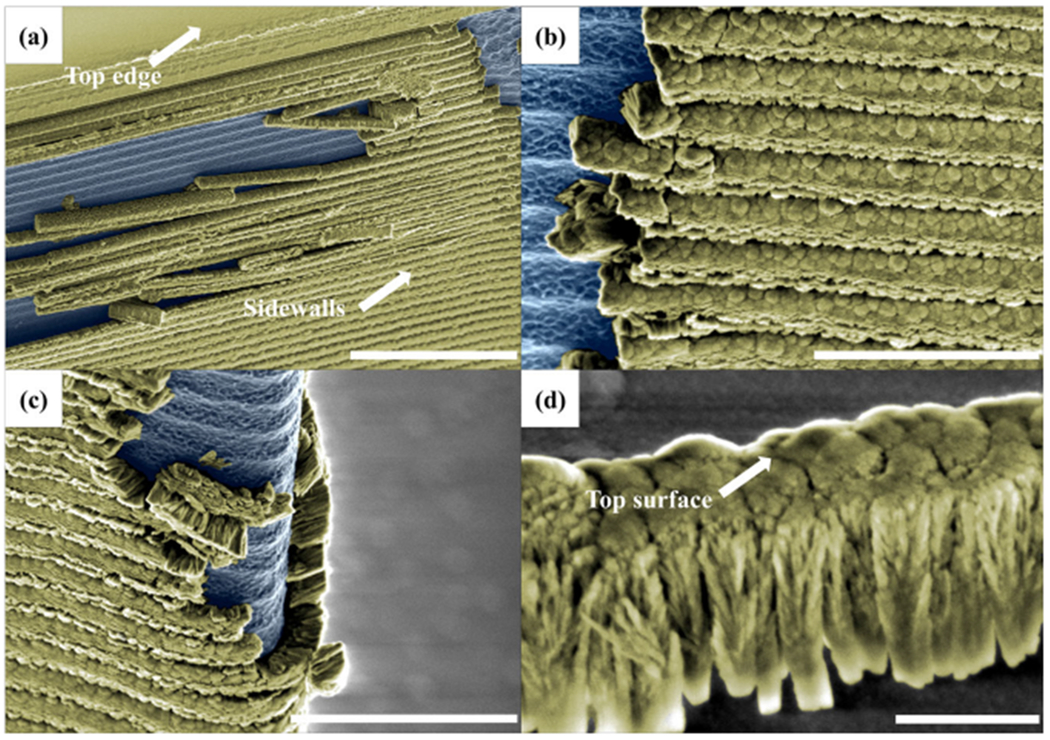
SEM images of an ALD/ICPCVD-protected device after 5 min KOH etching, showing silicon surfaces (blue), and SiO_2_ protection layers (yellow). **a** The protection layer collapsed in “strips,” starting from near the top. The scale bar is 10 μm. **b** The boundary between exposed silicon and SiO_2_ protection film shows an abrupt interface. The scale bar is 3 μm. **c** Closer view at the front edge of the device tip. The scale bar is 5 μm. **d** High magnification view of a dislodged SiO_2_ strip. The scale bar is 500 nm.

We investigated the ALD/ICPCVD dual-coating using SEM to determine why it performed only moderately well despite its higher overall film thickness and underlying ALD layer. Figure 3a shows the sidewalls after 5 min KOH etching which revealed strips of SiO_2_ corresponding to the periodic, scalloped morphology formed by the DRIE Bosch process on silicon. A higher magnification image (Fig. 3b) also supports the idea that SiO_2_ detached from the silicon surface in strips as opposed to being etched through. The detachment appears to start near the top, which was also seen at the tip of the device (Fig. 3c). A high magnification view of a fallen piece of ALD/ICPCVD SiO_2_ lying on its side (Fig. 3d) revealed the columnar growth structure of the film.

### FIB-SEM analysis of sidewall coverage

**Fig. 4:**
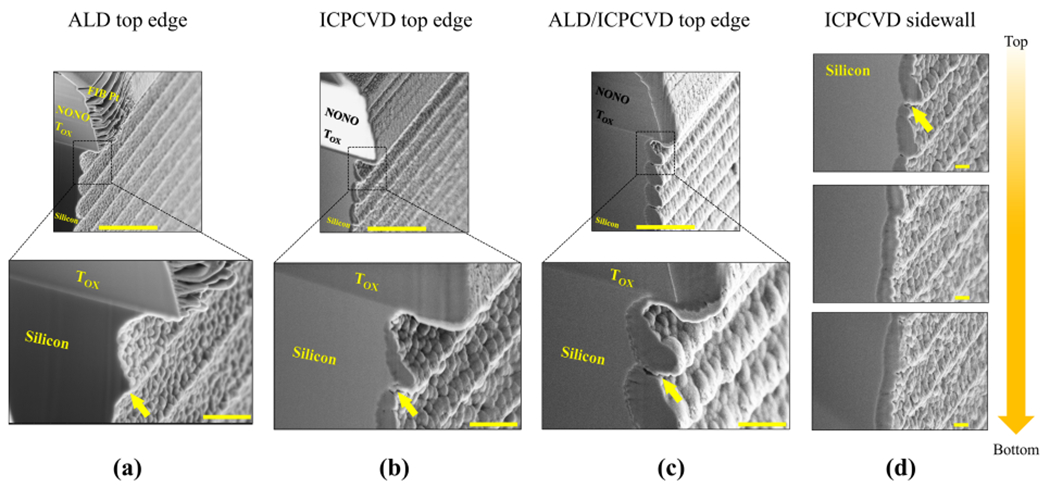
FIB-SEM cross-section views of sidewall coverage after deposition. Yellow arrows indicate the protection films and gaps in coverage. **a** Top edge of an ALD-protected device shows conformal and uniform coverage without gaps. The top and bottom images’ scale bars are 2 μm and 500 nm, respectively, for (**a-c**). **b** Top edge of an ICPCVD-protected device, in which low conformality is noted, and, in particular, gaps in coverage. **c** Top edge of an ALD/ICPCVD-protected device, in which a relatively uneven (bulging) film morphology and gaps in coverage are noted. **d** ICPCVD-protected sidewall, at various heights, revealing a noticeable gap in coverage at the top and more uniform coverage toward the bottom. The scale bars are 200 nm. T_OX_ = thermal oxide.

Figure 4a–c show cross-section views of the top edges of the devices before KOH etching for an ALD-protected, ICPCVD-protected, and ALD/ICPCVD-protected devices, respectively. All images show that silicon is slightly undercut laterally from the edge of the thermal oxide layer (T_ox_), a known effect of DRIE. ALD appears to conformally cover the sidewalls even at the hard-to-access area which is indicated by an arrow (Fig. 4a), in contrast to ICPCVD which leaves some gaps in the protection layer (Fig. 4b). These gaps become smaller further down the sidewall and the coverage eventually becomes continuous (Fig. 4d). This may explain why the sidewall protection collapsed from the top as seen in Fig. 1.

## DISCUSSION

Long-term reliability and biostability of silicon-based penetrating multisite microelectrodes and chemical, optical, or multimodal devices remains a major challenge^7^. An overlooked design consideration is to protect the exposed silicon sidewalls from the physiological environment. Exposed silicon sidewalls are present for the vast majority of devices reported so far^1,8–11,15–18^. This oversight may be a barrier to clinical approvals for human use. To the best of our knowledge, this is the first paper that systematically studied silicon sidewall dissolution in penetrating multisite microelectrodes as a potential failure source for chronic implantations. In this work, we developed batch-process-compatible sidewall protection strategies and assessed their coverage quality with a simple, rapid KOH etch.

Accelerated aging is the typical technique for evaluating neural microelectrodes’ long-term materials reliability. The elevated temperature of a phosphate-buffered saline solution exponentially accelerates aging of the device according to the Arrhenius equation^33^. However, the experiment may still take weeks to months depending on the desired lifetime of the implant in the human body. Assuming Arrhenius behavior, the rate of reaction approximately doubles with each 10 °C increase in temperature^34^. In other words, even at 87 °C, in order to study failure over an equivalent 20 years device lifetime in vivo, one would need to perform accelerated testing on the devices up to a period of 7.5 months in vitro. Reactive accelerated aging has been proposed as a more in vivo-like test by employing reactive oxygen species in addition to elevated temperatures, although it requires a specialized setup^35^. Our purpose in this study was to compare the quality of several SiO_2_ sidewall protection films. Our rapid KOH accelerated failure test reveals pinholes, defects, or unprotected areas in the SiO_2_ sidewall protection films due to its high etch selectivity for silicon. This rapid failure test can be used to evaluate insulation coverage faster than standard PBS-based accelerating soak testing and to comparatively screen candidate insulation films.

The 3D-extrapolated calculations of etched silicon volume in this study were made based on the assumption that the etched profile of the device’s front edge was consistent along the rest of the device’s sidewalls. It may be possible to construct more sophisticated 3D models using overlapping SEM images^36,37^. However, our focus was in obtaining relative performance metrics against the control.

Among the insulation materials tested, ALD performed the best in terms of surface coverage and quantified volume of etched silicon, as seen in Figs. 1 and 2, respectively. ALD’s uniform and conformal deposition property was crucial to its success. This property is due to its self-limiting surface reaction during film nucleation compared to conventional CVD techniques^38^. This feature enabled extremely conformal film growth which was critical for sidewall protection, particularly underneath the edge of the silicon shank (Fig. 4a). ICPCVD was too directional to cover all parts of the sidewall. We were not surprised to find that ALD/ICPCVD outperformed ICPCVD alone due to the presence of additional film thickness and the better coverage from the ALD film. However, its inferiority to ALD was intriguing. As both ALD and ICPCVD films are compressively stressed^39–42^, it is possible that the combined stress may have been large enough to cause the film to delaminate. It is also possible that the adhesion of ALD was stronger to ICPCVD (both are oxides) than to the silicon sidewall, contributing to delamination of the films (Fig. 3d). In both cases, deposition non-uniformity resulted in the bulging appearance near the top edge, as seen in Fig. 4c, which likely did not help sustain the sidewall structure.

**Fig. 5:**
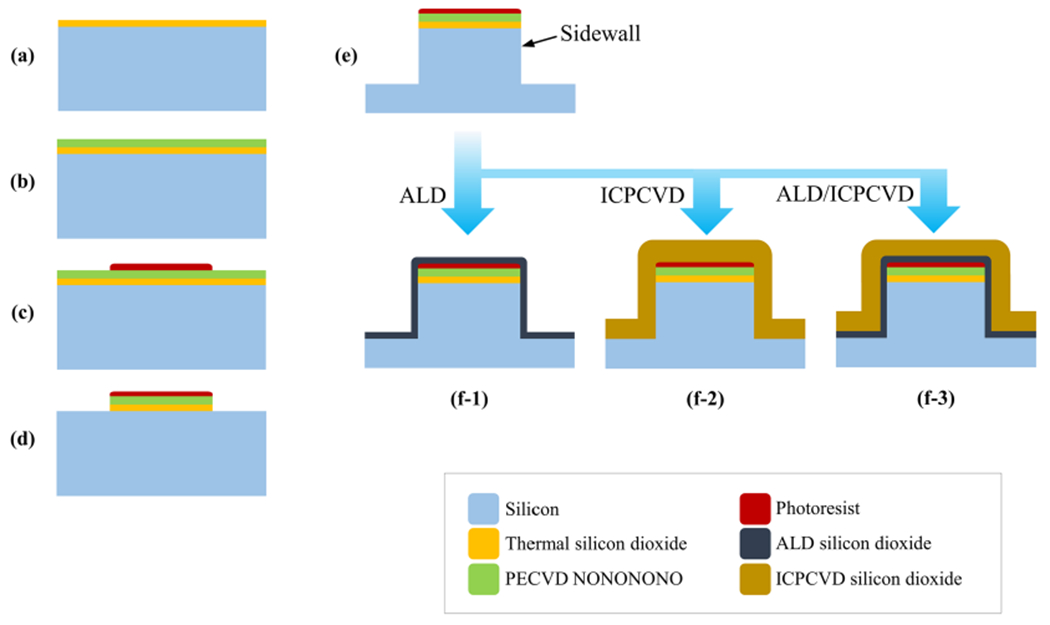
Fabrication flowchart showing the device’s cross-sectional views. **a** The starting wafer had a 1 μm-thick layer of thermal SiO_2_ over a 500 μm-thick silicon wafer. **b** SiO_2_/ SiN_x_ stack passivation layers were deposited by PECVD. Conductive traces were skipped for simplicity. **c** The shape of the devices was defined by photolithography. **d** Reactive ion etching selectively removed SiO_2_ and SiN_x_ layers. **e** Deep reactive ion etching of silicon shaped probe shanks, at which point the silicon sidewalls are exposed. **f** Conformal coating of silicon sidewalls was achieved by (**f-1**) ALD, (**f-2**) ICPCVD, or (**f-3**) ALD and ICPCVD. The photoresist was left in place during deposition in anticipation of functional device fabrication, in which case the photoresist would be needed to protect the electrode sites until the devices are released from the wafer.

In addition, we observed that failure of the sidewall protection started near the top edge (Fig. 5a) in all groups. We postulate that this is attributable to two mechanisms: (1) the protection films were less conformal near the top, exposing the silicon, and (2) better KOH diffusion access to the top areas may have caused faster etching of SiO_2_. FIB-SEM strongly suggests that the first mechanism played a role for ICPCVD films because there were easily-visible gaps in the protection layers near the top, but fewer near the bottom. These insulation gaps would have allowed KOH to etch silicon beneath the protection layers, causing them to detach. Meanwhile, ALD SiO_2_ did not show any visible gaps, but also showed a modest etching near the top after 30 min. This could be due to the second mechanism. Finally, it is possible that a combination of the two mechanisms played a role. The possibility of electrochemical corrosion as a contributing factor in this study is unlikely. These results provide an interesting contrast with a recent study that showed a bilayer of plasma-assisted-ALD Al_2_O_3_ and Parylene C failed more quickly than Parylene C alone. The suggested mechanism in this study was dissolution of the alumina film allowing undercutting and detachment of the Parylene C^43^. To avoid having aluminum in our ALD film, we performed a slower ALD process with trismethylaminosilane and ozone.

Our techniques for protecting sidewalls of a penetrating multisite microelectrode can be applied to most existing silicon-based devices. A technique for protecting functional devices is described below. Assuming the top feature plane is covered by hard-baked photoresist, which is the case after DRIE (Fig. 5e), a sidewall protection SiO_2_ film (e.g., ALD) can be deposited. A follow-up anisotropic dry etch can be performed to etch away the SiO_2_ film from the top and bottom horizontal surfaces, while leaving the vertical sidewalls mostly unchanged. In certain conditions, the thermal oxide projecting out over the sidewalls (Fig. 4a–c) would help protect the top corners of the sidewalls from the anisotropic etch. The rest of the process would be identical to what has been described in previous papers to backside etch and release probes from the wafer^12,13,20,44,45^.

In summary, most of the implantable silicon-based penetrating multisite neural devices leave the silicon sidewalls exposed to body fluids without protection. We investigated multiple SiO_2_ films as sidewall protection. We employed a KOH wet etch as a rapid evaluation of ALD and ICPCVD SiO_2_ film coverage quality. Among the studied groups, ALD provided the best coverage in terms of silicon etched volumes. Our results showed that ALD reduced etched silicon volume by a factor of 28.27 compared to bare silicon (control), while ALD/ICPCVD reduced it by a factor of 2.96, and ICPCVD by 1.99. We also showed that our rapid failure analysis technique is an effective engineering tool that allows expedited evaluation of film coverage. Application of this technique is not limited to microelectrodes and could be implemented in many types of silicon-based implantable devices requiring failure-free chronic implantation and FDA approval. Future work will compare these results with standard accelerating aging tests results and validate the ALD protection technique in a long-term in vivo functional study.

## METHODS

### Fabrication process overview

Figure 5 shows an overview of the microfabrication process which was part of a previously in vivo-validated device technology^12,13,44–48^. Four groups of devices were fabricated in this study: control (no protection), ALD-SiO_2_, ICPCVD-SiO_2_, and both ALD-and ICPCVD-SiO_2_. Briefly, the process began with a 100 mm-diameter silicon wafer which is heavily boron-doped, with resistivity 0.005–0.02 Ω·cm, and has 1 μm-thick thermal SiO_2_ (Ultrasil LLC, CA, USA) (Fig. 5a). Eight passivation layers were deposited in a plasma enhanced chemical vapor deposition (PECVD) system (STS, UK), in the order of NONONONO, where N is SiN_x_ and O is SiO_2_, to a total thickness of 1.85 μm (Fig. 5b). Then, to define the device shank, 9 μm-thick AZ4620 photoresist was patterned and hard baked at 100 °C for 30 min (Fig. 5c), followed by reactive ion etching (90 sccm Ar, 10 sccm C_3_F_8_) to etch through passivation layers and thermal oxide using an NLD-570 ICP RIE (ULVAC, MA, USA) (Fig. 5(d)). Deep reactive ion etching (DRIE) Bosch processing (400 sccm SF_6_, 1 sccm C_4_F_8_) in an Omega LPX Rapier DRIE system (SPTS, UK) was performed to etch approximately 80 μm into silicon, which exposed the sidewalls of the shanks (Fig. 5e). Then, except for the control sample, samples were coated with SiO_2_ using ALD and/or ICPCVD. Deposition details are provided in the following sections. Fabrication was performed at the University of Connecticut (CT, USA) and Harvard University (MA, USA).

### Atomic layer deposition (ALD)

ALD is known for highly conformal and pinhole-free film deposition at relatively low temperatures^38^. Approximately 50nm-thick SiO_2_ (1500 cycles) was deposited on the samples following DRIE (Fig. 5f-1). Trismethylaminosilane and ozone were used as precursors in an AT410 ALD system (Anric Technologies, MA, USA). The chamber temperature was 130 °C and the deposition rate was 0.02–0.035 nm per cycle. Film thickness was confirmed using ellipsometry (Film Sense LLC, NE, USA) on a witness silicon chip.

### Inductively-coupled plasma chemical vapor deposition (ICPCVD)

Inductively-coupled plasma can achieve higher film quality than conventional parallel electrode PECVD, even at lower deposition temperatures, due to its high-density plasma^39^. Approximately 1323 nm SiO_2_ was deposited on samples for 90 min in an ICPCVD reactor (Oxford Instruments, UK) (Fig. 5f-2). Film thickness was measured using ellipsometry on a witness silicon chip. The chuck temperature was 80 °C. Chamber gases were silane, helium, and oxygen. The ICP power was 1322 W. Residual stress was measured using a dual laser thin film stress measurement instrument (Toho Technology Inc, IL, USA) on a 100 mm-diameter silicon wafer before and after deposition (four repetitions).

### Accelerated failure test

We designed our etching experiment based on the well-established etch rate difference between silicon and SiO_2_ of two orders of magnitude^32,49^. Approximately 1.5 × 1.5 cm^2^ rectangular chips containing multiple dies from a 100 mm-diameter wafer were placed in 45% KOH in a beaker on a 60 °C hotplate. Three etching durations of 0min, 5 min, and 30 min were used. Samples were placed face-up at the bottom of the beaker and there was no agitation in the solution. Three devices were available in each group for analysis.

### Scanning electron microscopy

**Fig. 6:**
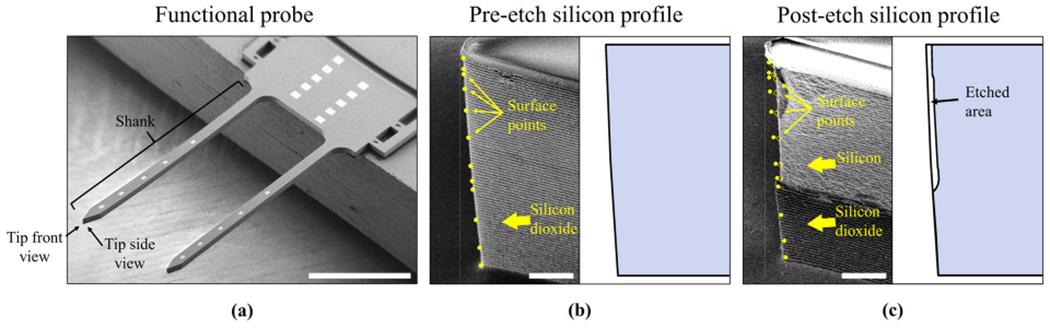
Overview of 3D extrapolation method based on SEM images. **a** A functional probe with an arrow indicating the SEM viewing direction (“tip side view”). The scale bar is 1 mm. **b** On the left is data collection from an SEM side view of the probe tip with sidewall surface points before KOH etch (solid circles). The scale bar is 10 μm. On the right is the side view of the generated model in SolidWorks based on those surface points. **c** Data collection from an SEM image after 30 min KOH etch with surface points (hollow circles). The scale bar is 10 μm. Right is the side view of the generated model in SolidWorks. The reduction in volume after 3D extrusion defined the etched volume.

Qualitative and quantitative evaluation of silicon sidewalls was conducted by SEM on each sample, before and after 30 min of KOH etching, using a SU8230 electron microscope (Hitachi High-Tech America, Inc., IL, USA). Devices were mounted and fixed horizontally on the SEM stage using carbon tape and were tilted 40°. Examples are shown in Fig. 6b, c. Detector mode was secondary electron, the working distance was approximately 14.5 mm, and the accelerating voltage was 2 kV. False coloring of the images was done in GNU Image Manipulation Program 2.10.

### 3D extrapolation and quantification

Quantification of etched silicon volume was achieved by 3D extrapolation of 2D SEM images. First, SEM images of shank tips (Fig. 6b-left) were imported into ImageJ^50^. Then, a series of points along the vertical edge of the shank were acquired and corrected to account for the 40° tilt as “surface points” (Fig. 6b-left). This step identified the surfaces of the silicon sidewalls. Separately, the design layout of the device (L-Edit, Tanner Research, USA) was imported into SolidWorks (Dassault Systèmes, France) and was extruded to the actual dimensions of the fabricated silicon device (Fig. 6b-right). Next, the surface points were used to define extruded cuts along all sidewalls of the model silicon shank, assuming etching was consistent along the lengths of the shank (Fig. 6b, c-right). Finally, the volumes of the etched and non-etched models were compared.

Statistical reliability was tested using one-way analysis of variance (ANOVA) with *N* = 3 for each group in Minitab19 (Minitab, LLC, IL, USA) followed by Tukey pairwise comparison for 95% confidence level. A *p*-value of 0.05 was considered statistically significant.

### Focused ion beam scanning electron microscopy

To understand the interior structures of the sidewalls, FIB cross-section images were taken. A 5 nm coating of 80:20 platinum:palladium alloy was sputtered onto the FIB samples to reduce charging. FIB cross sections were then obtained using a FEI Helios 660 (Thermo Fisher Scientific Materials and Structural Analysis Division, Oregon, USA) or ZEISS Crossbeam (Carl Zeiss Microscopy, LLC, NY, USA), both with gallium ion source. The cut was done on the edge of the device shank. A platinum strap was deposited by the FIB before cutting to protect the surface morphology of the sample.
